# Effects of ATLAS 2030 gait exoskeleton on strength and range of motion in children with spinal muscular atrophy II: a case series

**DOI:** 10.1186/s12984-022-01055-x

**Published:** 2022-07-19

**Authors:** C. Cumplido-Trasmonte, J. Ramos-Rojas, E. Delgado-Castillejo, E. Garcés-Castellote, G. Puyuelo-Quintana, M. A. Destarac-Eguizabal, E. Barquín-Santos, A. Plaza-Flores, M. Hernández-Melero, A. Gutiérrez-Ayala, M. Martínez-Moreno, E. García-Armada

**Affiliations:** 1 Centre for Automation and Robotics (CAR), Spanish National Research Council-Technical University of Madrid, Ctra. Campo Real km 0.2 – La Poveda-Arganda del Rey, 28500 Madrid, Spain; 2 International Doctoral School, Rey Juan Carlos University, Madrid, Spain; 3 Marsi Bionics S.L., Madrid, Spain; 4 Doctoral Program in Health Sciences, Alcalá de Henares University, Madrid, Spain; 5 Polytechnic University of Madrid, Madrid, Spain; 6 La Paz University Hospital, Madrid, Spain

**Keywords:** ATLAS, Exoskeleton, Children, Rehabilitation, Robot-assisted gait training, Spinal muscular atrophy, Strength, Range of motion

## Abstract

**Background:**

Children with spinal muscular atrophy (SMA) present muscle weakness and atrophy that results in a number of complications affecting their mobility, hindering their independence and the development of activities of daily living. Walking has well-recognized physiological and functional benefits. The ATLAS 2030 exoskeleton is a paediatric device that allows gait rehabilitation in children with either neurological or neuromuscular pathologies with gait disorders. The purpose is to assess the effects in range of motion (ROM) and maximal isometric strength in hips, knees and ankles of children with SMA type II after the use of ATLAS 2030 exoskeleton.

**Methods:**

Three children (mean age 5.7 ± 0.6) received nine sessions bi-weekly of 60 min with ATLAS 2030. ROM was assessed by goniometry and strength by hand-held dynamometer. All modes of use of the exoskeleton were tested: stand up and sit down, forward and backward walking, and gait in automatic and active-assisted modes. In addition, different activities were performed during the gait session. A descriptive analysis of all variables was carried out.

**Results:**

The average time of use was 53.5 ± 12.0 min in all sessions, and all participants were able to carry out all the proposed activities as well as to complete the study. Regarding isometric strength, all the measurements increased compared to the initial state, obtaining the greatest improvements for the hip flexors (60.2%) and extensors muscles (48.0%). The ROM increased 12.6% in hip and 34.1% in the ankle after the study, while knee ROM remained stable after the study.

**Conclusion:**

Improvements were showed in ROM and maximal isometric strength in hips, knees and ankles after using ATLAS 2030 paediatric gait exoskeleton in all three children. This research could serve as a preliminary support for future clinical integration of ATLAS 2030 as a part of a long-term rehabilitation of children with SMA.

*Trial registration*: The approval was obtained (reference 47/370329.9/19) by Comunidad de Madrid Regional Research Ethics Committee with Medical Products and the clinical trial has been registered on Clinical Trials.gov: NCT04837157.

## Introduction

Spinal muscular atrophy (SMA) is a severe neuromuscular disease characterized by the degeneration of the alpha motor neurons in the spinal cord, resulting in progressive proximal muscle weakness and paralysis [1]. SMA is the second most common fatal autosomal recessive disorder after cystic fibrosis, with an estimated incidence of 1 in 6000 to 1 in 10,000 live births [1].

Spinal muscular atrophy is phenotypically heterogeneous ranging from a life threatening to life altering disease, that is clinically classified into four phenotypes on the basis of age of onset and motor function achieved [2]. SMA type II is characterized by onset between 7 and 18 months of age. Patients achieve the ability to sit unsupported and some of them are able to acquire standing position. However, they do not acquire the ability to walk independently, and they usually live up to adolescence or longer [3].

In SMA patients, disease-related degeneration of spinal motor neurons results in reduced motor functions, muscle weakness, and atrophy [4]. This muscle weakness results as well in contracture formation, spinal deformity, limited mobility and activities of daily living (ADLs), increased risk of pain, osteopenia, and fractures. Weakness in these patients is usually symmetrical, and the proximal muscles at the scapular and the pelvic girdle are the most affected. Moreover, muscles of the lower extremities are weaker than those of upper extremities and the extensors are weaker than the flexors. Thus, joint deformities or flexums are often produced in the flexion position [5]. The use of orthoses to prevent these deformities or contractures and avoid limitation in ROM is common in children with SMA [6]. Resistance strength training exercise is recommended for these children [7] and it is known that motor function is directly linked to muscle strength, and that age-related loss of function in SMA is also related to loss of strength [8].

Robot-assisted gait training (RAGT) has been used over the past decade to help improve gait function [9]. This training provides conditions that support motor learning principles such as intensity, repetition, task specificity, and participation to promote both neuroplastic changes and non-motor recovery in patients with gait disorders [10]. In recent years, the literature on paediatric exoskeleton has increased significantly [11]. Several models of paediatric overground exoskeletons are found in the scientific literature in children with cerebral palsy [12, 13]. Benefits such as improved spatiotemporal gait parameters and energy consumption have been reported in this child population following the use of gait overground exoskeletons [12].

However, there is no literature that demonstrates the use of RAGT therapy in children with SMA [13], despite the benefits that have been reported in other childhood pathologies with gait impairments [12]. In addition, children with SMA who walk are significantly stronger than non-walkers [8]. Therefore, the use of robotic gait devices in children with SMA without walking ability could have numerous benefits at the musculoskeletal and functional levels.

ATLAS 2030 exoskeleton (Marsi Bionics S.L., Madrid, Spain) is the first overground gait exoskeleton CE marked for children with SMA and cerebral palsy. It consists of two robotic legs and trunk, and has eight degrees of freedom, four in each leg that allow hip, knee and ankle rotations in the sagittal plane, and hip rotation in the frontal plane. These actuators are based on ARES technology [14], which offers adjustable stiffness with the ability to minimise forces due to impacts, interacting safely with the patient while simultaneously storing and releasing energy in passive elastic elements. This adjustable and unique technology is crucial for children with SMA due to joint fragility, and it was specifically designed and intended for them [15]. As far as we know, there is no other paediatric exoskeleton with ARES technology. The ATLAS 2030 can be adapted to the anthropometric characteristics of each child by setting the geometry of the exoskeleton and the position of the cuffs used to attach the exoskeleton to the user's legs.

A proof-of-concept trial [16] in SMA children was conducted with the ATLAS 2025, the previous version, and research prototype, non-commercial of the current device, which showed the device was safe. The main differences between the ATLAS 2030 and its research prototype are in software upgrades, exoskeleton torso structure, battery and orthotics. In addition, ATLAS 2030 has been tested in children with cerebral palsy obtaining good results in ROM, strength and spasticity after the use of the exoskeleton [17].

As a newly developed device, there are no previous studies that have analysed ROM and strength changes in this population. Therefore, in order to evaluate the effects before and after using the ATLAS 2030 exoskeleton in a population of children with SMA type II, the main objectives of this work are: (1) to measure increase or decrease in the maximal isometric strength of the muscles groups responsible for lower extremity movement, and (2) to assess the changes in ROM of the hips, knees and ankles.

## Materials and methods

### Participants

Patients included in this case series study were recruited and assessed by a paediatric physician from La Paz University Hospital (Madrid, Spain) in order to ensure that they met the inclusion criteria of the study: Children from 3 to 14 years old, informed consent signed by the parents or legal guardians, the diagnosis of SMA type II, stable medical condition with no change in disease-specific medication in the last 6 months and no additional medication in the last month, being followed-up according to normal recommended standards for the disease, ability to maintain, spontaneously or with the help of a brace, the head and the trunk while standing and walking, no need for daytime ventilation, and inability to walk 10 m without aids, support or assistance. Exclusion criteria were: weight over 35 kg; femoral length (from hip joint to knee joint in the sagittal plane) smaller than 22 cm or larger than 38 cm, tibial length (from knee joint to ankle joint in the sagittal plane) smaller than 21 cm or larger than 37 cm; distance between trochanters smaller than 24 cm or larger than 40 cm; the inability to understand simple instructions; to report basic needs or to actively collaborate in the therapy; needing invasive of non-invasive daytime ventilation; Cobb angle higher than 25° without the possibility of wearing a brace during the test; severe skin alteration on the lower extremities, surgical intervention scheduled (spine, extremities) within the next 6 months, or surgery performed (spine, extremities) within the last 6 months; refusal of the patient or legal guardian to include the child in the study and skin problems (diseases, allergies, sensitivity, etc.) that prevent the use of the exoskeleton accessories on the patient's skin.

This study was performed in accordance with the Declaration of Helsinki [18] approval was obtained (reference 47/370329.9/19) by Comunidad de Madrid Regional Research Ethics Committee with Medical Products and all parents, or legal guardians, of the participants gave written informed consent. The clinical trial has been registered on Clinical Trials.gov: NCT04837157.

### Outcome measures

To monitor the training sessions, the total time of use of the exoskeleton and the duration of every activity was collected in minutes. To assess the improvements after the use of the exoskeleton, the maximal isometric strength of both lower limbs was measured using a Hand-Held Dynamometer microFET®2 (Hoggan Scientific, LLC., Salt Lake City, EEUU) (HHD) [5] following the methodology proposed by Bandinelli et al. [19]. The records were taken for each movement (hip flexion–extension, hip abduction–adduction, knee flexion–extension and anke dorsiflexion-plantarflexion). The HHD shows values in Newton (N) and is placed at the most distal possible part of the joint, in the opposite direction to the force requested from the participant.

Regarding the mobility test, all measurements are performed with the patient in supine position, a specialized physiotherapist used a manual goniometer and followed the rules provided by Norkin et al. [20]. Two types of mobility measurements were performed: (1) the degrees of joint limitation (flexums) in sagittal plane due to joint stiffness were measured in: hip extension, knee extension and ankle dorsal flexion. These measurements were taken in knee extension position; and (2) the full ROM of the hips, knees and ankles was measured in the sagittal plane [20].

The maximal isometric strength was measured three times for each movement and both legs. Before recording the strength values obtained, a test measurement was performed with all children to ensure that they had understood the instructions correctly and performed the correct movement.

Hip adduction was not measured as hip subluxation is commonly observed in children within this population.

### Device

**Fig. 1:**
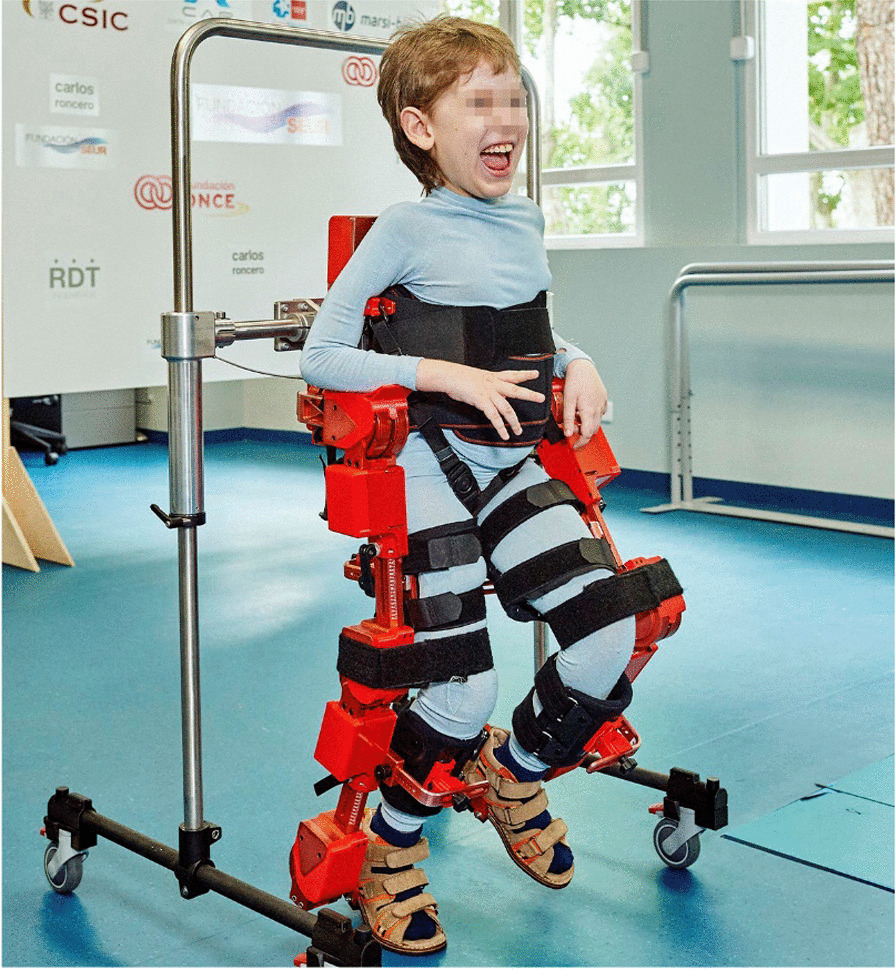
ATLAS 2030 exoskeleton

ATLAS 2030 main characteristics are defined in Table 1. ATLAS 2030 (Fig. 1) has been designed for children from 3 to 12 years old. Four degrees of freedom (DOF) drive each leg: three DOF at the hip, knee and ankle flexion–extension and one for abduction–adduction at the hip. The motion of the hip, knee and ankle joints is driven by rotational series elastic actuators, based on the ARES development [14].

**Table 1:** ATLAS2030 main characteristics

Mass	20.0 kg
Size adjustability	Thigh length (distance from the greater trochanter to the lateral condyle of the tibia) from 24 to 33 cm Leg length (distance from the lateral condyle of the tibia to the lateral malleolus) from 23 to 32 cm Pelvic width (between greater trochanters) from 24 to 35 cm
Joint torque	40 N*m (peak)
Gait velocity	0.1 m/s

The device is capable of walking forward and backward, providing walking assistance according to the user needs. It has two modes of action: (1) the automatic mode in which it assists the patient gait by totally following a gait pattern based on the kinematics of healthy subjects at the set speed and (2) the active-assisted mode in which the exoskeleton detects the intention of movement of the patient and assists finalising the step providing the remaining force needed. This intention to move is detected by force sensors placed on the device motors. In addition, the exoskeleton also allows movements from sitting to standing position. All these features are controlled through an application running on a tablet linked to the Wi-Fi connection provided by the exoskeleton itself. The main parameters can be changed using this App.

The control architecture of the device is divided in two interconnected systems [16]:

1. The main controller generates a synthetic trajectory through the information received from the user. This high-level controller is based on a real time processor, which calculate the inverse kinematic based on the parameters configured in the App and then the angle trajectory of each joint is sent to the motor low-level controller.
2. The low-level control systems receive the desired position and closes the loop with the position measured in each joint.

### Design of the study

The study was performed in Madrid at Marsi Care research facilities, located at the Centre for Automation and Robotics (CAR) from the Spanish National Research Council and Technical University of Madrid (CSIC-UPM). The study consisted of 10 sessions, organized as follows: one telephone screening visit (V0), one laboratory inclusion visit (V1), eight bi-weekly rehabilitation visits with the device (V2–V9), in which, in two of them (V5 and V9) the participants were also remeasured, and a final reassessment visit (V10).

During the first visit (V1), it was assessed that the participants did not meet any of the exclusion criteria that would prevent them from using the device, as well as the appropriate anthropometric measurements to be able to adjust the geometry of the ATLAS 2030 to the characteristics of the children (hip width, hip-to-knee distance and knee-to ankle distance). The first measurements of ROM and strength were taken, and a test of adjustment and adaptation to the device was carried out so that the children could adapt to its use.

Rehabilitation sessions (V2–V9) were biweekly every other day. The participants used the exoskeleton a maximum of 60 min to perform six activities in order to encourage the participation of young children in the study: (1) non-walking standing position (10 min); (2) sit-to-stand exercise using that mode of the exoskeleton (10 min); (3) walking forward and backward using both automatic and active-assisted modes of use of the exoskeleton (10 min in each mode); (4) trunk rotations while walking in automatic mode (10 min); (5) ball or balloon games while walking in automatic mode (5 min); and (6) balance exercises holding standing position (5 min). The walking area was 10 m × 6 m, providing the possibility to change directions to not only walk in a straight line. Rest periods were allowed if required by the participants. In all sessions using the device, the exoskeleton size was adjusted based on the anthropometric measurements of each patient: hip width, hip-to-knee distance and knee-to-ankle distance; and 15 min of lower limb muscle stretching was performed for the children’s comfort every session. Rest periods, device configuration and muscle stretching were not counted as exoskeleton usage time.

In training sessions V5 and V9, control measurements such as lower limb ROM and strength were collected at the beginning of each session. The last visit (V10) was performed 48 h after the last session with the exoskeleton, and to assess short-term changes that the use of the device may have produced on the ROM and strength.

### Statistical analysis

A descriptive analysis of all variables was carried out. The patient data were first analysed on a combined basis and secondly, in order to objectively measure the patient’s evolution, it was decided to evaluate the progression of the therapy by comparing each patient to himself by means of averages and standard deviations. All analyses and graphics were performed using IBM® SPSS® Statistics v27 software (IBM Corporation, Armonk, NY, USA) and Microsoft Excel 2019.

## Results

Due to SMA is considered a rare disease, so its prevalence is low [21–23], just four participants were assessed. However, one participant could not be included because he exceeded the maximum femur measurement allowed by the device (38 cm). Three patients (3 boys with a mean age 5.7 ± 0.6 years old, weight 22.0 ± 4.0 kg, and height 111.0 ± 2.6 cm) with SMA type II were recruited to participate in this study. Each patient´s description can be found in Table 2. None of the patients had walking ability and, therefore, they needed a wheelchair to move around. One of the participants (P1) used nightly BiPaP for prevention on alternate nights and used a Boston brace for scoliosis. Another participant (P2) had eventually participated in therapies with another treadmill exoskeleton (Lokomat®, Hocoma AG, Switzerland). All patients received Spinraza® treatment every 4 months from the age of two years (P2 and P3) and three years (P1).

**Table 2:** Patients’ description

Patient	Disease	FAC	Walking support	Age	Height (cm)	Weight (kg)
P1	SMA II	0	Wheelchair	6	114	22
P2	SMA II	0	Wheelchair	6	109	18
P3	SMA II	0	Wheelchair	5	110	26

All participants successfully completed the 10 sessions, and they used all exoskeleton´s modes of use and performed all the activities proposed. The average time of use per session was 53.5 ± 12.0 min. Of those, (1) 10.0 ± 0.0 min non-walking standing position; (2) 10.0 ± 0.0 min were spent performing the sit-to-stand exercise; (3) 19.0 ± 2.5 min walking in automatic and active-assisted modes of exoskeleton; (4) 10.0 ± 0.0 min doing trunk rotations while walking in automatic mode; (5) 4.6 ± 1.8 min doing balloon games while walking in automatic mode and (6) 4.7 ± 1.9 min performing balance exercises holding static position. Table 3 shows the layout of the session.

**Table 3:** Distribution of exoskeleton use during sessions V2 to V9 expressed in averages and standard deviations of each participant

Patient	Non-walking standing position	Sit-to-stand	Walking in automatic and active mode	Trunk rotations while walking	Balloon games while walking	Balance exercises holding static position
P1	7.8 ± 10.0	10.0 ± 0.0	18.0 ± 4.5	10.0 ± 0.0	6.0 ± 2.8	4.0 ± 0.0
P2	6.4 ± 2.2	10.0 ± 0.0	19.0 ± 2.0	10.0 ± 0.0	4.2 ± 0.5	5.2 ± 2.7
P3	8.4 ± 2.3	10.0 ± 0.0	20.0 ± 0.0	10.0 ± 0.0	4.0 ± 0.0	4.0 ± 0.0
Average	7.5 ± 2.1	10.0 ± 0.0	19.0 ± 2.8	10.0 ± 0.0	4.7 ± 1.7	4.4 ± 1.6

Based on the mean values in minutes of all sessions

**Fig. 2:**
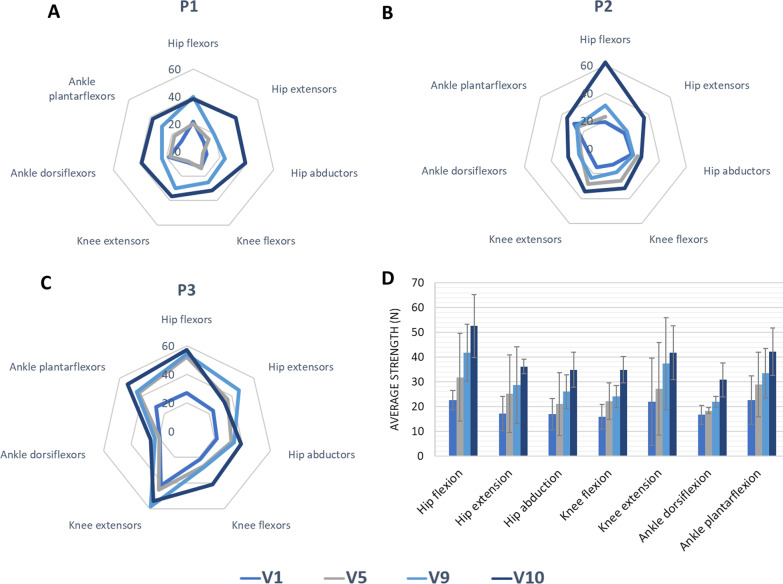
Average maximal isometric strength measurements collected for each patient (**A**, **B**, **C**) and for all the patients (**D**) for the different movements assessed in control visits (V1, V5, V9 and V10) measured with a Hand-Held Dynamometer in Newtons. P1, P2 and P3: Patient 1, 2 and 3. Error bars of **D** at 95% Confidence Interval (CI) show a standard deviation of the data

The maximal isometric strength measurements were collected from all participants (Fig. 2). The greatest improvements notable between the first five sessions (V1–V5) in flexor (60.2%) and extensor muscles (48.0%) of the hip, and plantar flexors of the ankle (35.4%). Overall, at the end of the study, all the measurements increased compared to the initial state, obtaining the greatest improvements for the hip flexion and extension movements and for the knee flexion (Fig. 2D). Moreover, in Fig. 2A–C a remarkable improvement of strength can be observed in the final visit V10, and during the previous two control visits, with respect to the initial visit V1 per patient for all the movements measured. As it can be observed, the hip extension strength from patient 2 visit 5 is missing since it could not be assessed due to minor pain in the participant’s hip extension movement. This did not interfere with the normal course of the session.

**Fig. 3:**
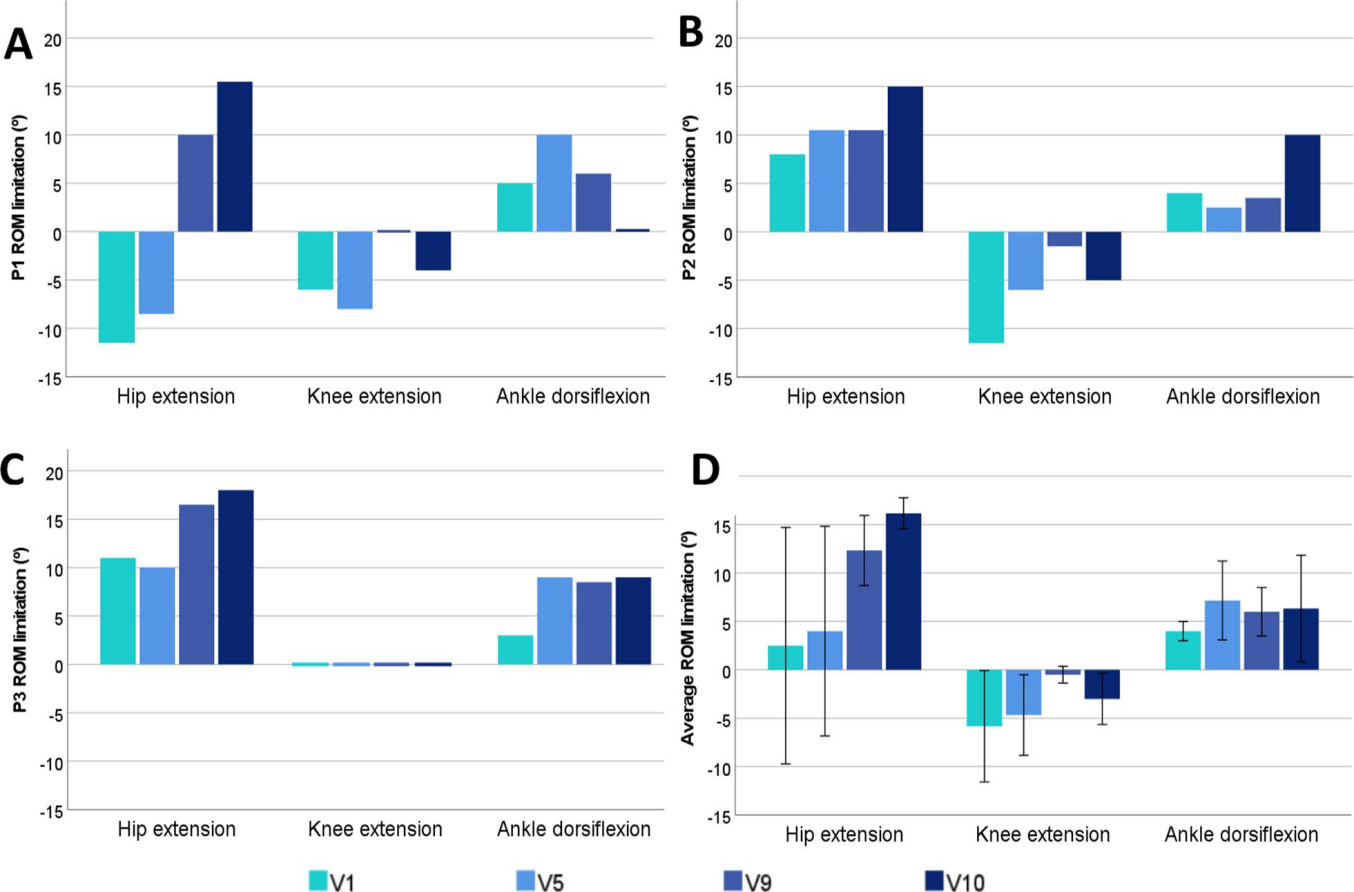
Progression of the degrees of joint limitation of hip extension, knee and ankle dorsiflexion in the different study participants (**A**, **B**, **C**). Graph **D** shows the average of all participants. Error bars of D at 95% CI show a standard deviation of the data. The lack of data visualisation for some measurements means that all values are at 0°

**Fig. 4:**
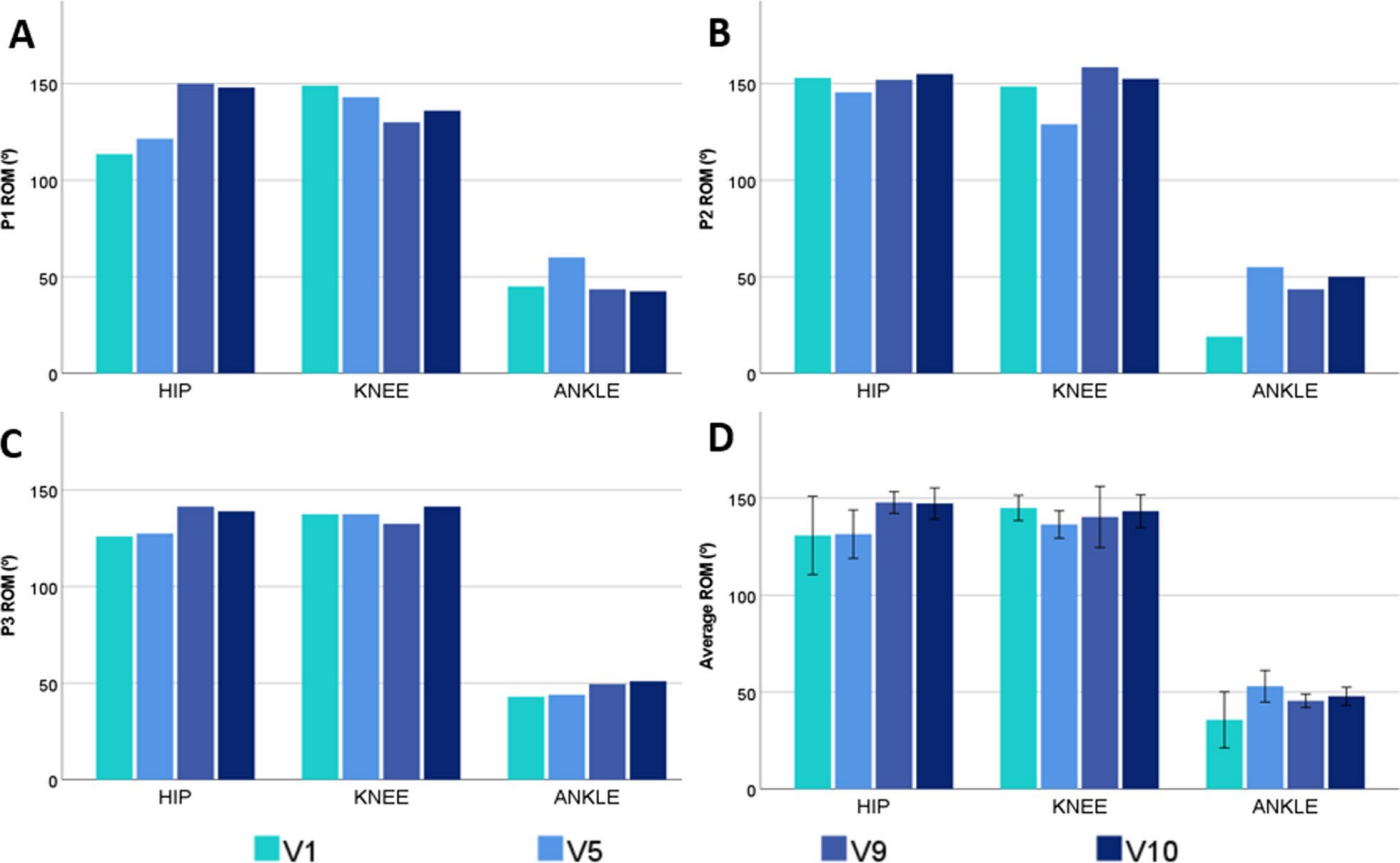
Progression of ROM at the hip, knee and ankle joints in the sagittal plane in the different study participants (**A**, **B**, **C**). Graph **D** shows the average ROM of all participants. Error bars of **D** at 95% CI show a standard deviation of the data

Regarding the movement limitation measurements or flexums, all of them (hip and knee extension and ankle dorsiflexion) improved after the use of the exoskeleton compared to the initial state (Fig. 3). Overall, the most remarkable improvements in knee extension and ankle dorsiflexion were observed in the first five exoskeleton sessions. In contrast, the greatest improvements in hip extension were observed between the fifth and ninth sessions. At the final assessment (V10) hip extension limitation increased the most with respect to baseline values (V1). Moreover, the ROM measurements (Fig. 4) increased 12.6% for the hip and 34.1% for the ankle in V10 with respect to V1. Regarding the knee ROM, it decreased a maximum of 5% in V5, but, finally, V1 value was maintained in V10.

## Discussion

The main objective of this work was to provide a first approach to the implementation of a novel robotic rehabilitation method that could help with the most important clinical aspects of the International Classification of Functioning, Disability and Health: Children and Youth Version (ICF-ICY) [24] framework for children with SMA type II. For this purpose, in this study, we examined the effects of ATLAS 2030 gait training in ROM and strength of these patients. All 3 participants who started the RAGT therapy completed it, with 100% compliance to scheduled RAGT sessions. This level of adherence is notable given the participants’ time commitment as well as the large amount of coordination needed between participants, therapist, and study team members [7].

Children with SMA present muscle weakness, atrophy and stiffness that results in a number of complications affecting their mobility, hindering their independence and the development of ADLs [4]. In this study, a remarkable improvement is obtained in the maximal isometric strength and ROM of lower limbs in children with SMA type II after attending RAGT therapy using the ATLAS 2030 exoskeleton for 9 sessions. It is worth noting that the measured strength for the hip flexion and extension movements reflected the highest improvement, just as it happened for the hip extension limitation. These improvements in hip strength could lead to a decrease in the probability of subluxation of this joint, which is so frequent in this population [25]. There is no literature that demonstrates the use of RAGT therapy in children with SMA, despite the benefits that have been reported in other childhood pathologies with gait disturbances [12, 13]. Therefore, the importance of this study is noteworthy as it is the first to evaluate the physical effects of a walking exoskeleton in children with SMA. In this regard, the ATLAS 2030 exoskeleton is the first paediatric gait exoskeleton originally intended for its use in children with SMA and no previous studies have been carried out to assess its efficacy. Thus, this is the first study to evaluate the physical improvements in maximal isometric strength and mobility of children with SMA type II after using ATLAS 2030 exoskeleton.

The results of the present study are difficult to compare with the scientific literature due to the lack of studies using exoskeleton for gait in children. Other studies have evaluated changes in maximal isometric strength exerted in children diagnosed with cerebral palsy. Bayón et al. [26] with CPWalker (CSIC, Spain) and Delgado et al. [17] with ATLAS2030 (Marsi Bionics, Spain) found improvements in strength after 8 weeks and 4 weeks of training with a walking exoskeleton, mainly showed important peaks of improvement for hip and knee flexion–extension, in line with our study. However, Kuroda et al. [27] with HAL® (Cyberdine, Japan) reported a decrease in knee extensors strength during 4-week training. Amman-Reiffer et al. [28] with Lokomat® (Hocoma AG, Switzerland) found no significant differences when comparing conventional treatment with ROM in cerebral palsy children. In our study, SMA participants obtained positive results in terms of ROM according with Zarkovic et al. [29] using Lokomat® Pro (Hocoma AG, Switzerland), however, knee ROM did not increase after the training sessions in our study. It should be noted that the studies cited above work with children with cerebral palsy, who may have spasticity which can interfere with comparing results between children with SMA and children with cerebral palsy, as children with SMA do not have spasticity.

ATLAS 2030 exoskeleton aims to increase the functionality, activities and participation of the children through the improvement of neuromusculoskeletal and movement-associated functions and mobility. Through the use of this device, and in accordance with the ICF, the functionality of children with SMA could be improved, due to the improvement in strength shown in these children. Specifically, interventions for the improvement of function, activities and participation such as lower limb strengthening, sit-to-stand training, standing posture maintenance, and gait training done with the exoskeleton, will be key to achieve the functional improvement [30]. However, along with the above-mentioned physical improvements, the fact of being able to walk and actively move using the exoskeleton favours the motivation and integration of children with disabilities [31].

Studies involving paediatric walking exoskeletons are mostly Non-Randomised Studies of Intervention (NRSIs) [13], due to the fact that these devices are not yet widely used in rehabilitation centres because of their novelty. For this reason, this study was a prospective pilot case series study with a number of potential limitations, such as including a small number of participants, the lack of a control group inherent in a pilot study and conducting the study for a limited period of one month. Because of these facts, it should be cautious to generalize the results of this study and, therefore, future studies should be conducted to assess the effectiveness of the device during a longer period of time and on a wider sample. However, since there has been no change in the treatment of the participants, the results found in the present study are thought to be due to the gait therapy with the ATLAS2030 exoskeleton.

## Conclusion

The improvements in ROM and maximal isometric strength of lower limbs achieved in this cases series study using ATLAS 2030 paediatric gait exoskeleton show the promising physical outcomes of RAGT therapy and the necessity of assessing functional changes in children with SMA type II. This research could serve as preliminary support for future clinical integration of ATLAS 2030 as a part of rehabilitation of children with SMA.

## Data Availability

The data supporting the conclusions of this article are included within the article and its additional files.

## Abbreviations

SMA: Spinal muscular atrophy
RAGT: Robot-assisted gait training
HHD: Hand-held dynamometer
ROM: Range of motion
ADLS: Activities of day living
CAR: Centre for Automation and Robotics
CSIC: Spanish National Research Council
UPM: Technical University of Madrid
FAC: Functional Ambulation Categories
CI: Confidence interval
ICF-ICY: International Classification of Functioning, Disability and Health: Children and Youth Version
NRSI: Non-Randomised Studies of Intervention
